# Establishment of an RAA-CRISPR/Cas12a assay based on *CpSge1* for rapid detection of *Cryphonectria parasitica*

**DOI:** 10.1128/spectrum.01079-25

**Published:** 2025-10-13

**Authors:** Haoyu Wu, Xiaorong Lin, Chengming Tian, Dianguang Xiong

**Affiliations:** 1 State Key Laboratory of Efficient Production of Forest Resources, Beijing Forestry University 12380, Beijing, China; 2 Beijing Key Laboratory for Forest Pest Control, Beijing Forestry University 659739 , Beijing, China; Pennsylvania State University, University Park, Pennsylvania, USA

**Keywords:** *Cryphonectria parasitica*, recombinase-aided amplification (RAA), CRISPR/Cas12a, lateral flow dipstick, rapid detection system

## Abstract

Chestnut blight disease caused by *Cryphonectria parasitica* is a serious branch disease that occurs worldwide, especially in Europe and North America. In recent years, chestnut blight disease has also been severe and even posed a great threat to the healthy development of chestnut orchards in some areas of China. Accurate and rapid detection of *C. parasitica* during the initial stages of the disease is helpful to take corresponding prevention and control measures in advance. In this study, we selected the *CpSge1* (Gti1/Pac2 transcription factor family) of *C. parasitica* as the detection target and established a rapid and visual detection system of *C. parasitica* that combined the recombinase-aided amplification (RAA) and CRISPR/Cas12a, called *CpSge1*-RAA-CRISPR/Cas12a. The system allows for the specific detection of *C. parasitica* in approximately 60 mins, with visualization of results. The detection sensitivity of this system was found to be 1 pg/µL. We combined the RAA-CRISPR/Cas12a with a lateral flow dipstick, which also showed specific, high sensitivity, and fast characters. In conclusion, the RAA-CRISPR/Cas12a assay has great potential to be a method for early diagnosis and on-site detection of *C. parasitica*, especially for areas where specialized equipment is lacking.

**IMPORTANCE:**

A rapid, highly sensitive, and visualized detection system of *Cryphonectria parasitica* was established by using the RAA-CRISPR/Cas12a method based on the C-terminal variable regions of a fungal-specific transcription factor *CpSge1*. The detection system was performed at a constant temperature condition of 37°C, which provides important support for the diagnosis of chestnut blight diseases in the field.

## INTRODUCTION

Chestnut blight is a devastating branch disease that causes significant damage to chestnut trees (belonging to the genus *Castanea*) worldwide, resulting in the death of numerous trees (1, 2). The causal agent, *Cryphonectria parasitica,* is a typical filamentous fungus that infects above-ground tree parts through wounds. Infected areas typically display necrotic lesions and exhibit rapid expansion, leading to the death of the bark and eventual death of individual trees or entire chestnut forests. In the late stages of the disease cycle, the characteristic orange-yellow stromata are formed on the surface of infected regions. These stromata serve to act as an important identified feature, containing a multitude of sexual or asexual spores. In recent years, chestnut forests in China have also been seriously attacked by *C. parasitica*, which has the potential to pose a threat to the healthy development of chestnut orchards in certain regions. Generally, chestnut trees at different growth stages can be infected by *C. parasitica* (3, 4). As for the management of chestnut blight, a range of control measures have been employed, including physical, chemical, resistant breeding, and biological control methods, which showed remarkable effects but could only mitigate the harm of chestnut blight. It is well documented that Cryphonectria hypovirus 1 (CHV1), a double-strand RNA virus, reduces the virulence of *C. parasitica*. Furthermore, CHV1 can spread to other *C. parasitica*, widely used as a biocontrol agent for chestnut blight in Europe (5, 6). Nevertheless, the effect of CHV1 in combating chestnut blight remains inadequate in other regions. Therefore, early detection and prevention are needed to reduce the harm of chestnut blight disease.

The Gti1/Pac2 transcription factor is a conserved fungal-specific protein family with a stable and conserved N-terminal domain (7, 8). However, their C-terminal regions are highly variable and not conserved among different fungal species (9). Generally, there are two members in this family, namely, Gti1/Sge1/Wor1/Rpy1/Mit1/Reg1/Fgp1 and Pac2, which show shared and distinct roles in different processes (10–16). Gti1/Sge1/Wor1/Rpy1/Mit1/Reg1/Fgp1, a conserved member of the Gti1/Pac2 protein family, plays important roles in fungal growth, sporulation, protein secretion, toxin synthesis, and pathogenicity, such as *Verticillium dahliae Sge1* (*VdSge1*) and *Zymoseptoria tritici Wor1* (white–opaque regulator 1) (*ZtWor1*) (17, 18). Previous studies have identified *CpSge1*, a member of the Gti1/Pac2 family, as a transcription factor that regulates the pathogenicity of *C. parasitica*. It was shown that *CpSge1* is the core regulator of fungal growth, stress tolerance, gene expression, and virulence in *C. parasitica*. Furthermore, the pathogenicity of the *CpSge1* deletion mutant was markedly reduced, demonstrating that *CpSge1* is a core gene governing the pathogenicity of this phytopathogen (19). Consequently, *CpSge1* was prioritized as the diagnostic target gene for *C. parasitica* detection, given its potential to enable species-specific molecular identification through conserved functional domains associated with fungal virulence regulation. This selection establishes a foundation for developing rapid diagnostic assays.

**Fig 1:**
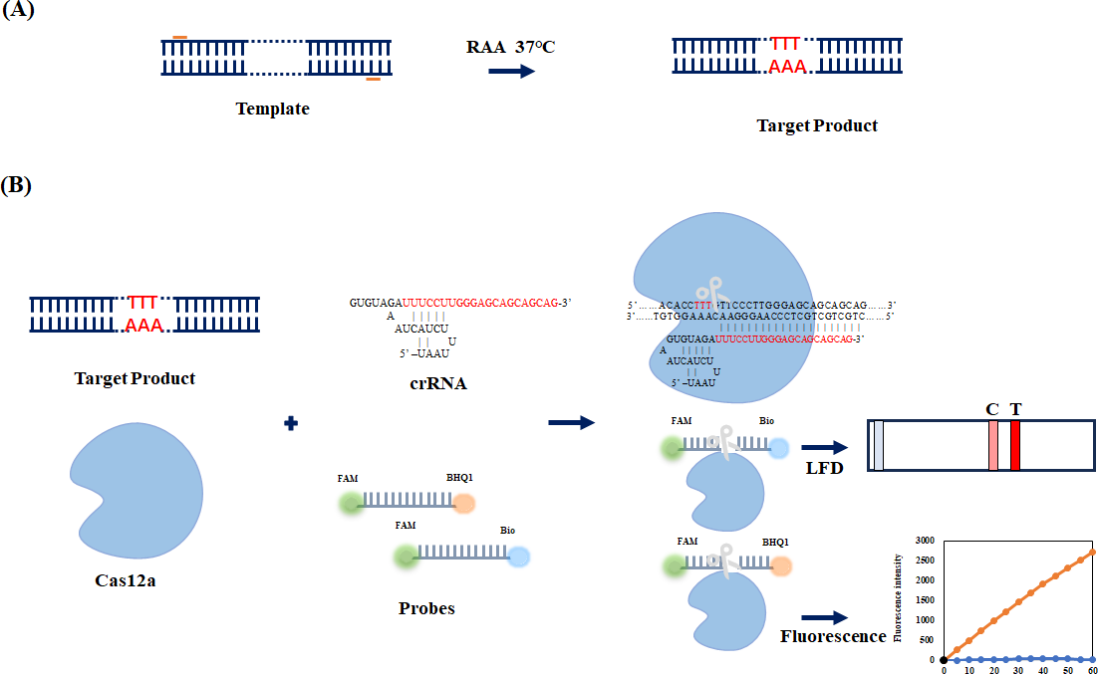
Schematic diagram of the RAA-CRISPR/Cas12a assay. (**A**) illustrates the RAA workflow, achieving exponential target DNA amplification under isothermal conditions. (**B**) illustrates the Cas12a/crRNA cleavage assay workflow and fluorescence-based detection (quantitative) and the LFD assay for dual validation of CRISPR-Cas12a activity. The visual bands on the test strip typically comprise a control line (C line, which serves to validate the detection validity) and a test line (T line, indicating the presence of the target analyte). The C line must exhibit visible coloration to confirm result reliability, while the intensity of the T line coloration correlates with the concentration of the target analyte.

In recent years, the high sensitivity and good specificity of the CRISPR/Cas system have made it widely used in the detection of various pathogens, especially the CRISPR/Cas12a system (20, 21). Cas12a, also known as Cpf1, can cut non-targeted single-stranded DNA (ssDNA), which is an RNA-directed monomeric endonuclease protease belonging to the Class 2 (Type V) CRISPR/Cas systems (22, 23). The CRISPR/Cas12a system can detect amplification products containing the correct protospacer adjacent motif (PAM; 5′-TTTN-3′) sequence, thereby reducing false-positive signals and significantly improving the specificity of the assay (24). Cas12a and crRNA were incubated to form ribonucleoprotein (RNP) complexes. These complexes bind to the target DNA by recognizing the 5′-TTTN-3′ PAM sequence (located 3′ to the protospacer) through crRNA guidance, followed by cleavage of the target double-stranded DNA (dsDNA) (25). Upon activation, Cas12a exhibits collateral cleavage activity, non-specifically cleaving nearby single-stranded DNA (ssDNA) probes in the solution (26, 27). The detection results can be rapidly interpreted via fluorescence readout, which is suitable for both laboratory and field detection. When integrated with isothermal amplification methods, including recombinase polymerase amplification, recombinase-aided amplification (RAA), and loop-mediated isothermal amplification (LAMP), the CRISPR/Cas12a system enables highly specific and sensitive nucleic acid detection (28, 29). RAA is an isothermal nucleic acid amplification technology that operates at a constant temperature (e.g., 37–42°C) and requires minimal equipment, significantly reducing reliance on expensive experimental equipment and consumables (30). The RAA-CRISPR/Cas12a system leverages dual-layer specificity: (i) sequence-specific amplification by RAA and (ii) PAM-dependent target recognition and crRNA-guided cleavage by Cas12a (21). The results can be interpreted through fluorescence readout (e.g., using a portable blue light transilluminator for FAM-labeled probes) or lateral flow dipstick (LFD) assays (e.g., via FAM/biotin-labeled reporters), enabling flexible readout options for both laboratory and field detection (31, 32). The detection process diagram is shown in Fig. 1. The RAA reaction exponentially amplifies the target DNA under isothermal conditions, achieving sufficient template concentration for downstream detection. Upon completion of amplification, the CRISPR/Cas12a RNP complex binds to the amplified DNA via crRNA-guided hybridization and PAM recognition, inducing site-specific dsDNA cleavage. This target-activated Cas12a further exhibits collateral cleavage activity, non-specifically degrading fluorophore-quencher-labeled ssDNA probes (e.g., FAM-BHQ1) or dual-labeled reporters (e.g., FAM/biotin-ssDNA), thereby generating a measurable signal (fluorescence or lateral flow readout). By the aforementioned principle, the fluorescence-quenched ssDNA probe was employed as a fluorescent reporter. The Cas12a system uses a fluorescent ssDNA probe (FAM-BHQ1) for detection. Target-activated Cas12a cleaves the probe, turning on fluorescence. For field use, a dual-labeled probe (FAM/biotin) enables lateral flow strips to show color changes (positive: two lines; negative: one line). This combines CRISPR precision with rapid visual readouts (33, 34).

Therefore, this study applied the RAA-CRISPR/Cas12a assay for rapid detection of *C. parasitica*, establishing a theoretical foundation for developing targeted strategies to control its spread and develop its ecological impact.

## MATERIALS AND METHODS

### Extraction of strains and genomic DNA

*C. parasitica*, *Colletotrichum gloeosporioides*, *Verticillium dahliae*, *Cytospora chrysosperma*, and *Pseudocryphonectria elaeocarpicola* were used in our detection and specificity assays were performed and maintained in our laboratory. *Gymnosporangium yamadai* was collected from the infected apple leaves and used for DNA extraction during its biotrophic feature. The genomic DNA (gDNA) of each strain was extracted by the CTAB method and stored at −20°C.

### Construction of *CpSge1*-T-vector

The sequence of *CpSge1* was acquired from the reference genome of *C. parasitica* EP155 in the NCBI database (https://www.ncbi.nlm.nih.gov/). Then, the gDNA of *C. parasitica* was used as the template, and the primers CpSge1-F and CpSge1-R were used to amplify *CpSge1*. The reaction solution includes 2× SanTaq PCR Master Mix (Sangon Biotech [Shanghai] Co., Ltd.‌‌), CpSge1-F and CpSge1-R primers, DNA template, and water to a volume of 50 µL. The resulting products were purified using the TIANgel Purification Kit (Tiangen Biotech [Beijing] Co., Ltd., Beijing, China), resulting in the full-length fragment of the *CpSge1*. The purified *CpSge1* fragment was ligated to the T-Vector pMD19 (Takara Bio Inc.), and the *CpSge1*-T-vector was acquired.

### Design of primers and crRNA

The sequences were searched using the NCBI database, which included the following organisms: *C. parasitica, C. chrysosperma, Fusarium graminearum, Zymoseptoria tritici, Fusarium verticillioides, V. dahliae, Magnaporthe oryzae,* and *Botrytis cinerea*. The sequence alignment was performed using Jalview. Two crRNAs were designed from the C-terminal variant region of *C. parasitica*. According to the recognition site of crRNA, primers for RAA were designed. Two candidate primers for the RAA assay were designed by following the design principles of RAA primers. The primers, crRNAs, and marked probes were synthesized by Beijing Tsingke Biotech Co., Ltd. The RAA amplification reaction was performed by following the instructions of the RAA Nucleic Acid Amplification Kit (Jiangsu Qitian Gene Biotechnology Co., Ltd., Jiangsu, China). The primers and the probes used are listed in Table 1.

**TABLE 1:** Sequence of PCR primer, RAA primer, crRNA, and probe

Name	Sequence	Purpose
CpSge1-F	ATGGACGAGCTTGTGTCGCA	Amplification of the full length of CpSge1
CpSge1-R	AAGGACGCGAAGAACTGATCG	
Cp-crRNA1	UAAUUUCUACUAAGUGUAGAUUUCCUUGGGAGCAGCAGCAG	Guide RNA1
Cp-crRNA2	UAAUUUCUACUAAGUGUAGAUCCGACAUGAUGCUGUUGGCC	Guide RNA2
CpRAAF	TCCTGGACGACCGAGCCCTACTCCTTGGAATGA	Amplification of CpSge1 RAA fragment 1
CpRAAR	CTGCTGCTGCTGCTGCTGTTGGTGGTGTTG	
CpRAA2F	GAGCGGACATCCTCCCTACCACAGATACCACAGA	Amplification of CpSge1 RAA fragment 2
CpRAA2R	TCTTCTCTTGCTGCTGCTGCTGGTGCTGCT	
ssDNA1	5′-FAM-TTATT-BHQI-3′	Probe 1
ssDNA2	5′-FAM-TTTTTTTATTTTTTT-biotin-3′	Probe 2

### Construction of RAA-CRISPR/Cas12a assay of *C. parasitica*

The RAA-CRISPR/Cas12a detection system contains two parts: the RAA reaction and the CRISPR/Cas12a reaction. First, the gDNA or *CpSge1*-T-vector of *C. parasitica* was used as a template for RAA reactions conducted with two primer pairs, respectively, for optimal primer selection using the RAA nucleic acid amplification basic kit as per the manufacturer’s instructions. A mixture containing 25 µL of buffer V, 14.5 µL of purified water, 2 µL of primer F (10 µM), and 2 µL of primer R (10 µM) was manually mixed, briefly centrifuged, and added to the basic reaction unit. Following thorough mixing and brief centrifugation, 5 µL of magnesium acetate and 1.5 µL of gDNA were added; the mixture was vortexed and incubated in a preheated 37°C metal bath for 40 min, and the RAA amplification product was obtained. Then, the reaction system consists of 1× NEBuffer 2.1 (New England Biolabs [Beijing] Ltd.), Cas12a protein (50 µM; New England Biolabs [Beijing] Ltd.), crRNA (50 µM), ssDNA1 (50 µM), amplification product, and DEPC water. Then, the system was incubated at 37°C for 1 h in the Light Cycler 96 System, and the final system was exposed to the ultraviolet. Furthermore, the ssDNA1 probe was replaced with ssDNA2 to align with the Cas12/13 special nucleic acid strip (Tiosbio Inc.) with the CRISPR/Cas12a reaction system. The system was incubated at 37°C for 40 min. Subsequently, the reaction product was diluted 10-fold, with 5 µL added to 45 µL of water. The FAM/Bio dual-labeled amplicons served as the detection targets. The reaction products were applied to the lateral flow strip and incubated for 2 min at room temperature, enabling rapid on-site detection of *C. parasitica*. The test line appears when gold-conjugated anti-FAM antibody binds to FAM label on the ssDNA. Biotin label is captured by streptavidin immobilized on the test line. The presence of both the test line and the control line indicated a positive test result. Conversely, a negative test result was indicated by the presence of only the control line. In the absence of the control line, the test was deemed invalid.

### Optimization of CRISPR/Cas12a assay

To optimize the CRISPR/Cas12a reaction system, we conducted the RAA-CRISPR assay using *C. parasitica* gDNA and the selected optimal RAA primers and probe within a concentration range of Cas12a (1 µM, 0.5 µM, and 0.25 µM) and crRNA (1 µM, 0.75 µM, 0.5 µM, and 0.25 µM), respectively. Finally, the optimum crRNA, concentration of Cas12a, and crRNA were confirmed and selected for further analysis.

### Specificity and sensitivity of the RAA-CRISPR/Cas12a assay for *C. parasitica* detection

To confirm the specificity of the RAA-CRISPR/Cas12a assay developed for detecting *C. parasitica*, *C. gloeosporioides, G. yamadai, P. elaeocarpicola, V. dahliae,* and *C. chrysosperma* were analyzed. The results were then visualized using the RAA-Cas12a-LFD assay.

To determine the sensitivity of the RAA-CRISPR/Cas12a assay for *C. parasitica* detection, different concentrations of gDNA (1,000 ng/µL) and *CpSge1*-T-vector (100 ng/µL) were tested. A 10-fold gradient dilution was performed continuously, extending from 1 × 10^−8^ to 1 × 10^0^ to obtain the required concentration of gDNA and *CpSge1*-T-vector. The gDNA and *CpSge1*-T-vector were employed as templates for the RAA-CRISPR/Cas12a assay. The copy number was also calculated. Subsequently, the results were visualized using the RAA-Cas12a-LFD assay.

### Diagnosis of field samples using the developed RAA-CRISPR/Cas12a assay

To ensure the reliability and applicability of the RAA-CRISPR/Cas12a assay for *C. parasitica* detection in the field, we tested healthy and infected *C. parasitica* into chestnut branches. Nuclease-free water was used as a negative template control (NTC). Five-millimeter mycelial plugs from PDA plates of *C. parasitica* strains were placed onto the scorched areas and secured with sealing film. Inoculated branches were maintained at room temperature under high humidity for 20 days. Total DNA was extracted from the bark of healthy and infested branches at the spots. Primers CpSge1-F/R were used to amplify a 1,626-bp fragment within the *CpSge1* gene for verification. The extracted total gDNA was analyzed using the RAA-CRISPR/Cas12a assay for visual detection of the results. Three replications of each experiment were made.

## RESULTS

### Design and optimization of detection primers and crRNA

**Fig 2:**
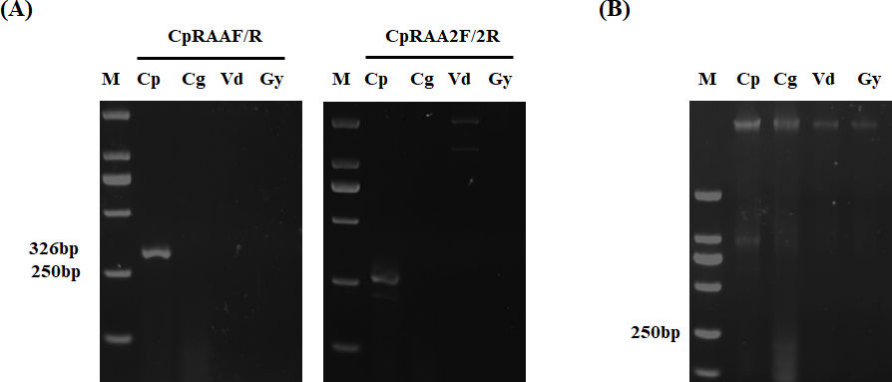
The optimal amplification primer pairs screening using RAA. (**A**) Detection of RAA amplification products. (**B**) Verification of gDNA from four fungal pathogens. Lane M: 250 bp DNA marker; gDNA from four fungal pathogens—*C. parasitica* (Cp), *C. gloeosporioides* (Cg), *V. dahliae* (Vd), and *G. yamadai* (Gy)—was amplified by RAA using specific primer pairs CpRAAF/R and CpRAA2F/2R. The size of the amplification product of CpRAAF/R is 326 bp.

**Fig 3:**
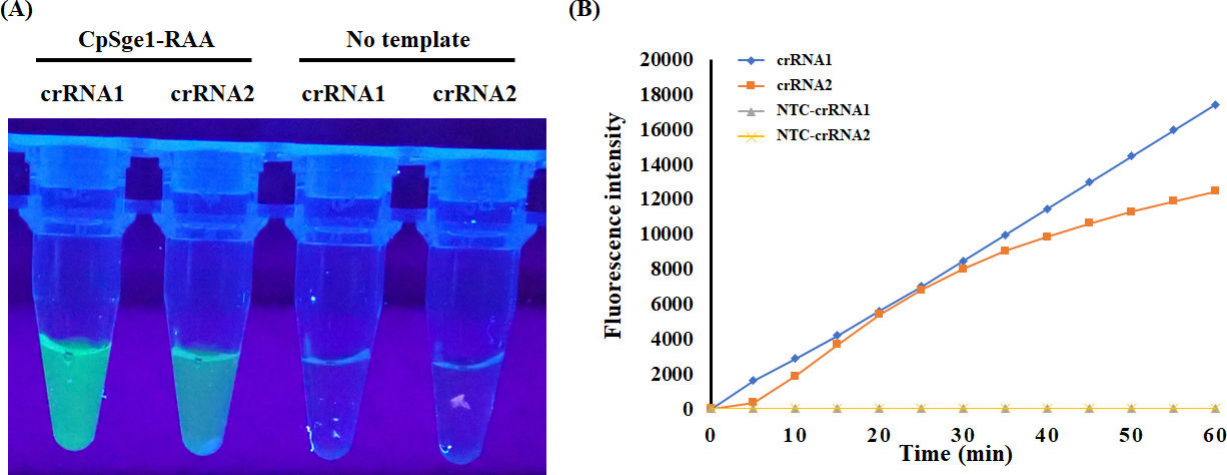
crRNA design optimization for RAA-CRISPR/Cas12a specificity enhancement. (**A**) Fluorescence imaging of Cas12a-mediated cleavage activity on RAA-amplified *C. parasitica* gDNA using crRNA1 or crRNA2. (**B**) Real-time fluorescence kinetics quantifying Cas12a activation efficiency under optimized crRNA conditions.

As mentioned above, the Gti1/Pac2 transcription factor family, which generally consists of two members: Gti1 (also known as Sge1, Wor1, or Ryp1) and Pac2, is specific in fungi. The N-terminal Gti1/Pac2 domains are highly conserved among the Gti1 orthologs from different fungal species, while the C-terminal regions are various, as shown in Fig. S1. Therefore, we selected the C-terminal regions of *CpSge1* as the target for detection. Following the guidelines of RAA primer designing, two sets of primer pairs, CpRAAF/R and CpRAA2F/2R, were manually designed from the *CpSge1* gene of *C. parasitica*. To evaluate the specificity of the designed two primer pairs, the RAA amplification was carried out using *C. parasitica*, *C. gloeosporioides, G. yamadai,* and *V. dahliae* as templates. Notably, the RAA products produced by the CpRAAF/R primer pair exhibited the highest intensity and specific band. CpRAAF/R was identified as the optimal choice due to its superior performance in terms of non-specific amplification and amplicon length (Fig. 2). Subsequently, two crRNAs were separately used to detect *C. parasitica* in the CRISPR/Cas12a assay (Fig. S1). The probe was cleaved when the amplified product was mixed with the different components of the CRISPR/Cas12a assay, and a fluorescent signal was released in the presence of the *CpSge1*-T-vector and the Cas12a/crRNA complex. As shown in Fig. 3, the obvious fluorescent signal was observed and detected for the *CpSge1*-T-vector samples, while no fluorescent signal was detected for the negative control. Additionally, the fluorescence intensity of the reaction products by using crRNA1 as the guide was notably stronger than that of crRNA2 (Fig. 3A), and the fluorescence value generated by using the crRNA1 showed a well-linear upward trend, and the peak value of crRNA2 was lower than that of crRNA1 (Fig. 3B). The results suggested that the crRNA1 showed a better effect than the crRNA2 in the CRISPR/Cas12a assay. Thus, crRNA1 was chosen for the subsequent experiment. Figure S2 is the target sequence of crRNA for the detection of *C. parasitica*.

### Optimization of the CRISPR/Cas12a assay

**Fig 4:**
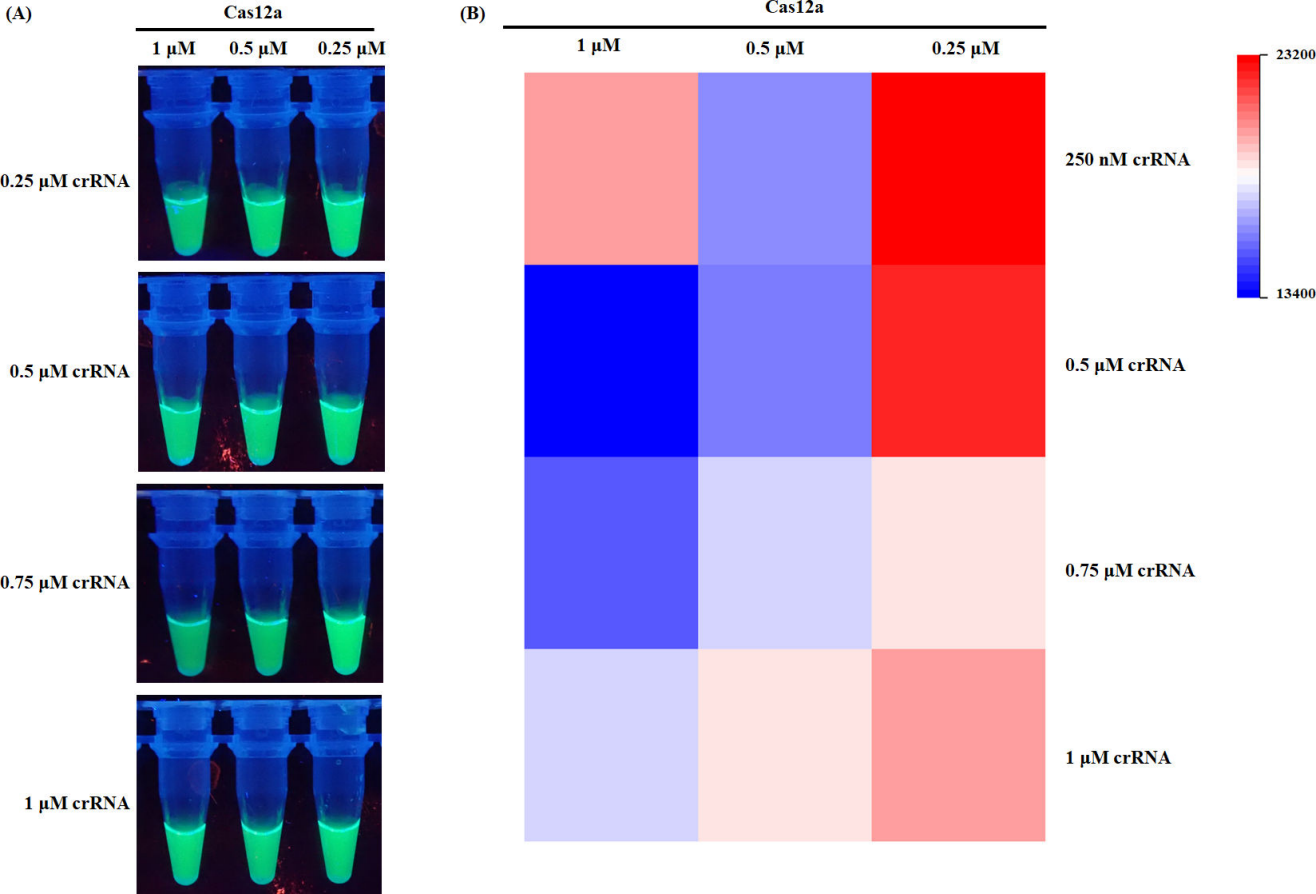
Optimization of the Cas12a and crRNA concentration. (**A**) Endpoint fluorescence imaging (UV transillumination, 365 nm) of Cas12a-mediated cleavage across 12 combinatorial conditions (Cas12a: 0.25, 0.5, and 1 µM; crRNA: 0.25, 0.5, 0.75, and 1 µM). (**B**) Heatmap with optimal activity at 0.25 µM Cas12a + 0.25 µM crRNA.

In this study, we tested the optimization of the CRISPR/Cas12a assay. To this end, Cas12a (1, 0.5, and 0.25 µM) and crRNA1 (1, 0.75, 0.5, and 0.25 µM) were tested at different concentrations. Four combinations of Cas12a and crRNA1 displayed high fluorescence values, including 1 µM Cas12a and 0.25 µM crRNA1, 0.25 µM Cas12a and 0.25 µM crRNA1, and 0.25 µM Cas12a and 0.5 µM crRNA1, and 0.25 µM Cas12a and 1 µM crRNA1 (Fig. 4). Excessively high concentrations of Cas12a may increase non-specific cleavage activity, whereas insufficient concentrations can impair its ability to efficiently cleave the target DNA, thereby compromising the overall cleavage efficiency of the system. An excessively high concentration of crRNA1 may lead to non-specific binding interactions, while a suboptimal concentration may inadequately facilitate the targeting of Cas12a to the desired locus, thereby compromising the precision and efficacy of the CRISPR-Cas12a system. In consideration of reagent cost, we selected 0.25-µM Cas12a protein and 0.25-µM crRNA1 for the CRISPR/Cas12a assay in further experiments. Three replications of each experiment were made, and the results were highly consistent across all replicates.

### Specificity of the CRISPR/Cas12a assay of *C. parasitica*

**Fig 5:**
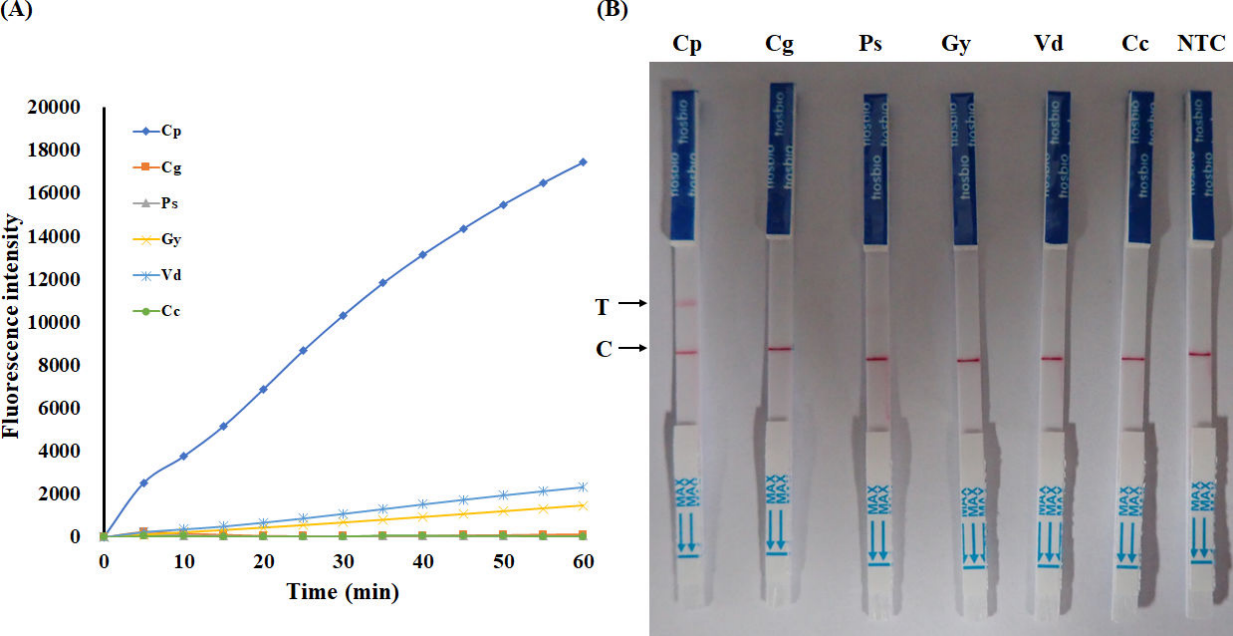
Specificity evaluation of the RAA-CRISPR/Cas12a system. (**A**) Real-time fluorescence kinetics comparing Cas12a-mediated cleavage activity across gDNA from six phytopathogens: *C. parasitica* (Cp), *C. gloeosporioides* (Cg), *P. elaeocarpicola* (Ps), *G. yamadai* (Gy), *V. dahliae* (Vd), and *C. chrysosperma* (Cc). (**B**) Test strips show a distinct T-line signal exclusively for *C. parasitica* gDNA, confirming the high specificity of the RAA-CRISPR/Cas12a system. C line indicates valid assay procedure.

For the specificity evaluation of the CRISPR/Cas12a assay, six fungal species were selected: *C. parasitica* and the other five fungi. Among these, *C. parasitica* and *C. gloeosporioides* were known to be associated with chestnut tree disease, while the remaining four species served as negative controls. As shown in Fig. 5, by RAA combined with CRISPR/Cas12a, *C. parasitica* and other species were clearly distinguished by fluorescence visualization and fluorescence signal values (Fig. 5A). The dipstick results demonstrated that only *C. parasitica* exhibited both the test line and the control line, indicating that the CRISPR/Cas12a detection system exhibited excellent specificity for *C. parasitica* (Fig. 5B). The results indicated that both the fluorescence detection and the RAA-CRISPR/Cas12a-LFD methods of the *C. parasitica* CRISPR/Cas12a assay exhibited high specificity. Three replications of each experiment were made, and the results were highly consistent across all replicates.

### Sensitivity of CRISPR/Cas12a assay of *C. parasitica*

**Fig 6:**
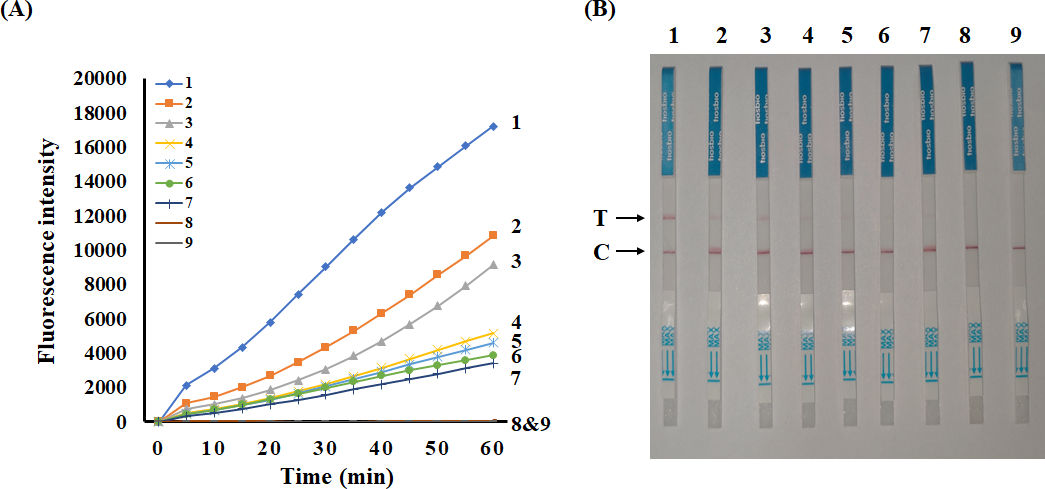
Sensitivity profiling of the RAA-CRISPR/Cas12a system for *C. parasitica* gDNA detection. (**A**) Real-time fluorescence kinetics showing the Cas12a-mediated cleavage activity across a dynamic range of *C. parasitica* gDNA. A significant linear correlation was observed between the fluorescence intensity and the logarithm of *C. parasitica* gDNA concentration. The fluorescence detection achieved a lower limit of 1 pg/µL, and the copy limit for detection is 2.1 × 10^1^ copies/μL. (**B**) LFD dipstick assay validation of sensitivity thresholds. Test strips display visible T-line signals down to 1 pg/µL, and the copy limit for detection is 2.1 × 10^1^ copies/μL. The C line indicates valid assay operation. The gDNA concentrations for samples 1–9 follow a 10-fold serial dilution series: 10^3^, 10^2^, 10^1^, 10^0^, 10^−1^, 10^−2^, 10^−3^, 10^−4^, and 10^−5^ ng/μL. The total DNA amounts are 2,000 ng, 200 ng, 20 ng, 2 ng, 200 pg, 20 pg, 2 pg, 0.2 pg, and 0.02 pg, respectively.

**Fig 7:**
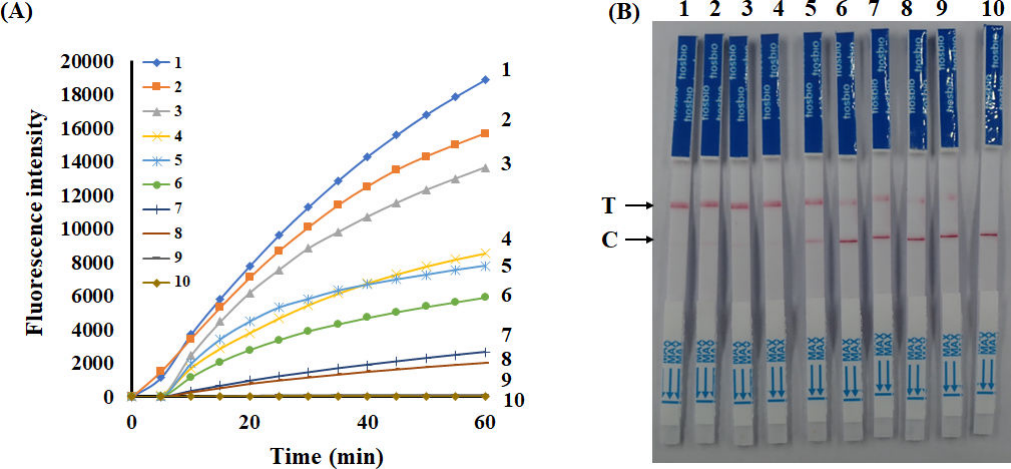
Sensitivity profiling of the RAA-CRISPR/Cas12a system for *CpSge1*-T-vector detection. (**A**) Real-time fluorescence kinetics showing Cas12a-mediated cleavage activity across a dynamic range of *CpSge1*-T-vector. A significant linear correlation was observed between the fluorescence intensity and the logarithm of *CpSge1*-T-vector concentration. The fluorescence detection achieved a lower limit of 10 fg/μL, the *CpSge1*-T-vector concentration is 1 × 10^−8^ µg/µL, and the copy limit for detection is 2.32 × 10^3^ copies/μL. (**B**) Lateral flow dipstick assay validation of sensitivity thresholds. Test strips display visible T-line signals down to 10^−8^, the *CpSge1*-T-vector concentration is 1 fg /μL, and the copy limit for detection is 2.32 × 10^2^ copies/μL. The C -line indicates valid assay operation. The *CpSge1*-T-vector concentrations for samples 1–9 follow a 10-fold serial dilution series: 10^2^, 10^1^, 10^0^, 10^−1^, 10^−2^, 10^−3^, 10^−4^, 10^−5^, and 10^−6^ ng/μL, sample 10 is no temple control. The total *CpSge1*-T-vector amounts are 200 ng, 20 ng, 2 ng, 200 pg, 20 pg, 2 pg, 0.2 pg, 0.02 pg, 0.002 pg, and 0, respectively.

To determine the sensitivity of CRISPR/Cas12a of *C. parasitica*, the gDNA of *C. parasitica* (1,000 ng/µL), *CpSge1*-T-vector (100 ng/µL), and their continuously 10-fold gradient diluted samples (10^0^ to 10^−8^) were used as the template, respectively. The test results showed that when the gDNA was diluted 10^−6^ (Fig. 6A and B), the fluorescence signal could be detected and two clear bands were observed, which were indicated by the strong color signal at the test line on the LFDs. However, when the DNA concentration was further decreased, the test lines disappeared. Therefore, the limited concentration of gDNA detected by the RAA-CRISPR/Cas12a assay was 1 pg/µL, and the limited detected copy number was 2.1 × 10^1^ copies/μL (Fig. 6). Furthermore, fluorescent signals could be detected when the *CpSge1*-T-vector was diluted to 10^−7^. Upon further dilution to 10⁻⁸, two clear bands were observed (Fig. 7A and B). Therefore, the limited concentration of *CpSge1*-T-vector was 1 fg/μL, and the limited detected copy number was 2.32 × 10^2^ copies/μL by using the RAA-CRISPR/Cas12a-LFD system. The findings indicated that the RAA-CRISPR/Cas12a detection assay was susceptible to *C. parasitica*. Experiments were independently conducted four times, and the results were reproducible.

### Actual sample testing

**Fig 8:**
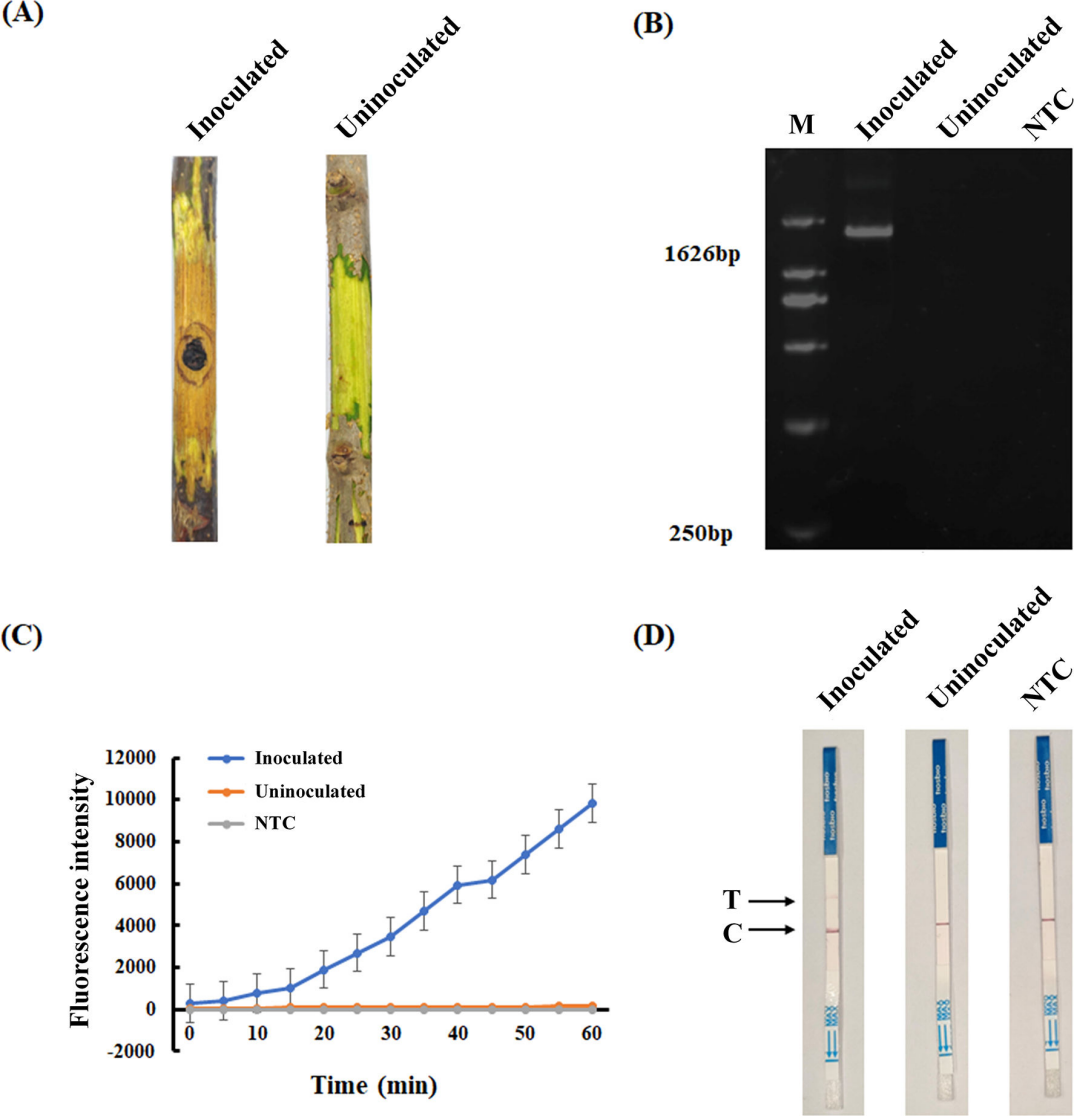
Clinical sample detection of *C. parasitica* using the RAA-CRISPR/Cas12a system. (**A**) Phenotypical comparisons of inoculated and uninoculated plant branches. (**B**) Molecular validation of inoculated and uninoculated plant branches. PCR products amplified from uninoculated and inoculated branches with CpSge1-F/R. The size of the *CpSge1* is 1,626 bp. (**C**) Real-time fluorescence kinetics demonstrating Cas12a-mediated cleavage activity in clinical samples. Inoculated chestnut tissues exhibited rapid fluorescence amplification, while uninoculated chestnut tissues showed minimal background signals. (**D**) LFD assay results. Distinct T-line signals were observed exclusively in inoculated samples, with C lines confirming valid assay operation. The NTC contained nuclease-free water in place of template DNA.

To evaluate the performance of the RAA-CRISPR/Cas12a assay, healthy chestnut branches and chestnut branches infected by *C. parasitica* were tested using the RAA-CRISPR/Cas12a assay established in this study. Healthy one-year-old chestnut branches were selected and sterilized with a flamed steel rod to create a 5-mm-diameter wound. Subsequently, a 5-mm-diameter mycelial plug from a 7-day-old *C. parasitica* culture was aseptically inoculated onto the wounded site. gDNA was separately extracted from both *C. parasitica*-inoculated branches and non-inoculated healthy branches. The infected branches exhibited characteristic brown necrotic lesions, contrasting with the asymptomatic phenotype of the non-inoculated healthy controls (Fig. 8A). PCR analysis with primers CpSge1-F/R amplified a 1,626-bp product specific to *C. parasitica* in the infected sample. Absence of this amplicon in the healthy sample and the negative control confirmed pathogen infection (Fig. 8B). Then, the non-inoculated healthy sample and the inoculated sample were used for RAA amplification and CRISPR/Cas12a reaction. The results showed that fluorescence signals could only be observed or detected in infected chestnut branches (Fig. 8C). Meanwhile, both the test line and the control line were observed (Fig. 8D), indicating that both the fluorescence detection method and the LFD method could detect *C. parasitica* in practical applications. Each experiment was conducted in triplicate with consistent results.

## DISCUSSION

*C. parasitica* seriously affects the ornamental, ecological, and economic value of chestnut, and the occurrence of the disease has not been effectively controlled (35). Once the hosts are infected by *C. parasitica*, the necrotic lesions quickly surround the branches and trunks, causing canker symptoms, which significantly reduce the yield and quality of chestnuts, and may even lead to the death of the entire tree (36). Identifying and detecting *C. parasitica* based on field symptoms would easily lead to misdiagnosis and missed diagnosis. Conventional methods for detecting *C. parasitica* rely mainly on observing symptoms and identifying the morphology of the pathogen (37). These methods often require specialized equipment, such as sophisticated thermal cyclers and skilled technicians. The conventional molecular biological detection methods are contingent upon expensive instruments and apparatus, and the steps are complex and time-consuming, which presents a challenge in meeting the needs of rapid on-site detection (38, 39). Thus, it is crucial to establish new assays to detect the presence of *C. parasitica*.

Classical molecular biological detection methods (such as duplex PCR) exhibit significant limitations in detecting *C. parasitica*. This approach requires two specific primers and achieves target amplification through 30–40 thermal cycles. The complete detection process typically takes 60 min and is susceptible to interference from non-specific amplification, resulting in false positives and background amplification. Compared to traditional PCR, RAA employs a recombinase-single-stranded DNA-binding protein system to achieve nucleic acid amplification under isothermal conditions. This method not only significantly reduces reaction time but also eliminates reliance on precision thermal cyclers, thereby markedly enhancing the feasibility of on-site pathogen detection (40). This enables it to better meet the requirements of rapid and simple on-site pathogen detection (41). Both RAA-CRISPR/Cas12a-LFD and LAMP systems achieve detection within approximately 1 h. However, RAA operates under isothermal conditions without requiring precise thermal cycling, significantly simplifying instrumentation compared to LAMP (typically requiring 60–65°C) (42). This feature enables field deployment using portable heating blocks. RAA primers targeting the *C. parasitica* ensure target-selective amplification. CRISPR/Cas12a’s collateral cleavage activity is activated only upon crRNA hybridization to amplified products, eliminating non-specific signals through orthogonal validation. The CRISPR/Cas12a diagnostic system achieves single-molecule detection sensitivity through crRNA-guided specific recognition and trans-cleavage activity. The lateral flow detection readout method is particularly suited for resource-limited settings, enabling visual interpretation without instrumentation. The CRISPR/Cas12a diagnostic systems have been widely implemented in detecting diverse pathogens (43), which do not rely on complex equipment and show high sensitivity, making them ideally suited for rapid diagnostic scenarios in the field (41, 44).

Our assay demonstrated no cross-reactivity with the aforementioned pathogens. False-positive results may occur due to contamination by other pathogenic fungi in the environment. Despite the availability of several laboratory and commercial assays for the detection of *C. parasitica* in chestnut, there remains a need for rapid and accurate detection methods that are suitable for field applications. The selection of target genes allowed the RAA-CRISPR/Cas12a method to have good specificity. The sensitivity of the real-time PCR method for the detection of *C. parasitica* in bark tissues was 2 fg of gDNA (45). In this study, the limit of detection of the RAA-CRISPR/Cas12a assay was determined to be 1 pg/µL, which was more sensitive than the conventional end-point PCR method. This indicated that RAA-CRISPR/Cas12a could offer an earlier identification of infection than PCR, thereby providing a potential advantage in disease prevention. Furthermore, the RAA-CRISPR/Cas12a assay successfully detected *C. parasitica* from chestnut branches. This suggests that it was a viable option for practical, non-laboratory diagnosis in the monitoring of *C. parasitica* infection.

Furthermore, compared to cumbersome sampling processes and time-consuming laboratory deliveries, on-site diagnostics offer significant advantages in terms of convenience and time efficiency. The reaction does not necessitate thermal cycling and can be conducted with only limited equipment support, such as a portable mini-centrifuge and a set of pipettes. It can be accomplished by employing the RAA reaction combined with the on-site DNA extraction method; based on the UV flashlight irradiation, the fluorescence was distinguishable with 2.1 × 10^1^ copies/μL of the gDNA. Similarly, in the detection combined with LFD, the sensitivity could reach a minimum of 2.1 × 10^1^, and the results could be judged directly through the eyes. These methods facilitate rapid on-site inspection by reducing equipment requirements, inspection time, and cost through visual inspection using a portable UV flashlight and Cas12/13 special nucleic acid strip. Although our method has certain advantages, it is not without limitations. It is worth noting that the RAA amplification process may have inadvertently increased the risk of aerosol contamination and false-positive results due to the necessity of opening the lids of the reaction tubes for product transfer. The objective of our ongoing study is to overcome this limitation, which includes the development of non-opening operations and an increase in the size of samples.

In conclusion, a rapid detection system was developed that enables real-time monitoring in the chestnut growing environment. This method does not require complex, large-scale instrumentation or specialized personnel and is capable of rapidly and accurately identifying *C. parasitica* in chestnut plants. The developed system showed great potential in rapid field detection of C. *parasitica* in infected chestnut orchards.

## SUPPLEMENTAL MATERIAL

Fig. S1 spectrum.01079-25-s0001.tif Alignment of the CpSge1 sequence.

Fig. S2 spectrum.01079-25-s0002.tif Schematic of crRNA design targeting CpSge1 for RAA-CRISPR/Cas12a specific detection.

Supplemental material spectrum.01079-25-s0003.docx Supplemental figure legends.

ASM does not own the copyrights to Supplemental Material that may be linked to, or accessed through, an article. The authors have granted ASM a non-exclusive, world-wide license to publish the Supplemental Material files. Please contact the corresponding author directly for reuse.
