# Supplementary figures and images for: Establishment of an RAA-CRISPR/Cas12a assay based on CpSge1 for rapid detection of Cryphonectria parasitica
Source: Microbiol Spectr. 2025 Oct 13;13(11):e01079-25. doi: 10.1128/spectrum.01079-25 (PMC12584721; doi:10.1128/spectrum.01079-25)

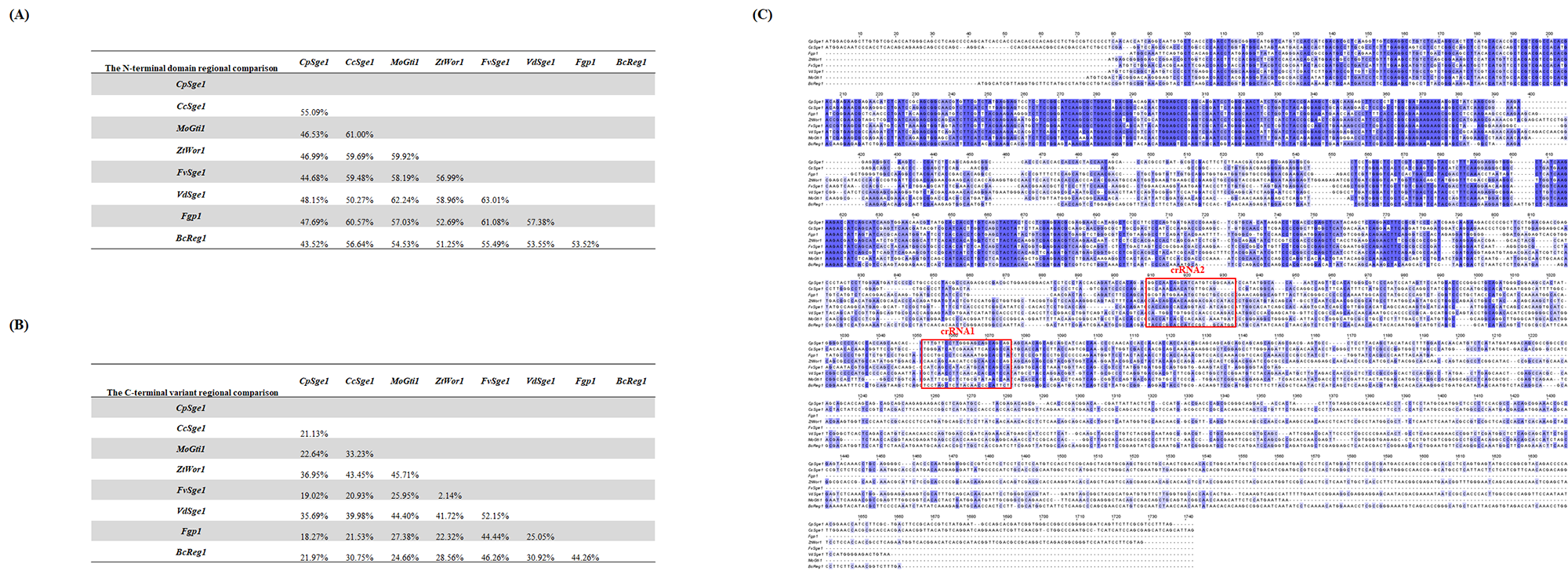

Supplement: Fig. S1 — Alignment of the CpSge1 sequence. [file spectrum.01079-25-s0001.tif]

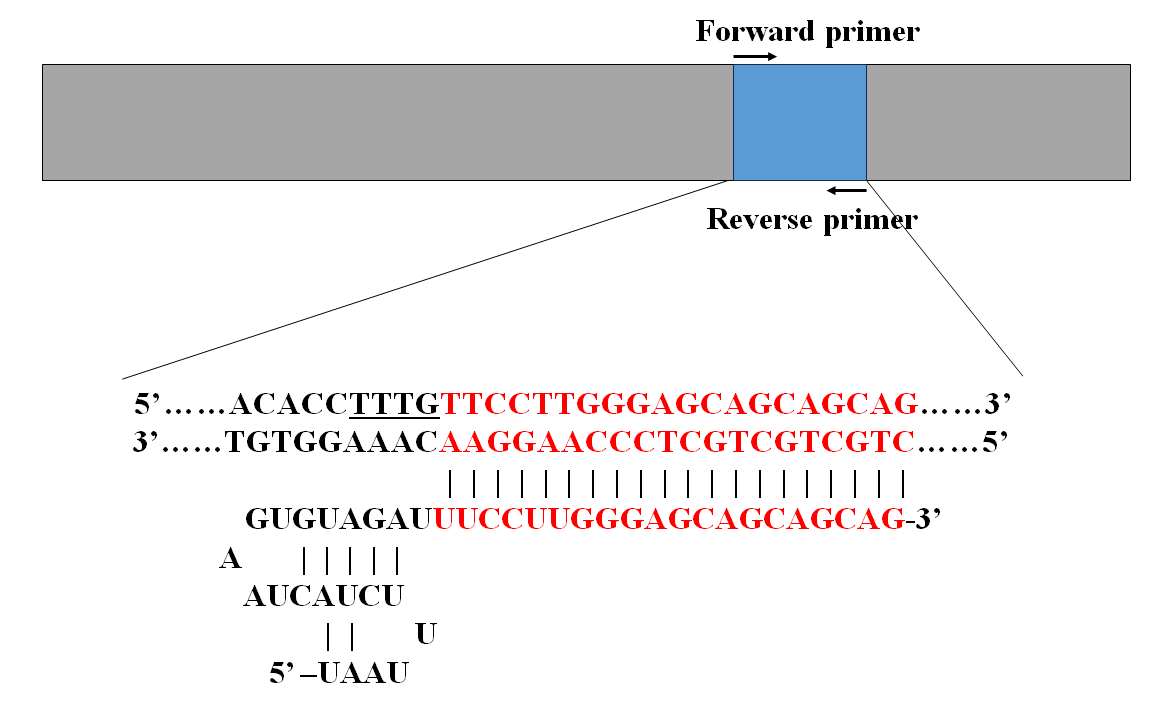

Supplement: Fig. S2 — Schematic of crRNA design targeting CpSge1 for RAA-CRISPR/Cas12a specific detection. [file spectrum.01079-25-s0002.tif]
