# Supplementary material for: Establishment of an RAA-CRISPR/Cas12a assay based on CpSge1 for rapid detection of Cryphonectria parasitica
Source: Microbiol Spectr. 2025 Oct 13;13(11):e01079-25. doi: 10.1128/spectrum.01079-25 (PMC12584721; doi:10.1128/spectrum.01079-25)
Supplement: Supplemental material — Supplemental figure legends. [file spectrum.01079-25-s0003.docx]

**Figure S1** Alignment of the *CpSge1* sequence. **A** is the result of the N-terminal domain regional comparison. **B** is the result of the C-terminal variant region comparison. **C** is the site of two crRNAs. *The CpSge1* sequence of the *C. parasitica* strain was used for BLASTN alignment in the NCBI database. *Cpsge1*: *Cryphonectria parasitica, CcSge1*: *Cytospora chrysosperma, Fgp1*: *Fusarium graminearum, ZtWor1*: *Zymoseptoria tritici, FvSge1*: *Fusarium verticillioides, VdSge1*: *Verticillium dahlia, MoGti1*: *Magnaporthe oryzae, BcReg1*: *Botrytis cinerea* is the gene name of the sequence source genome in the NCBI database. The alignment part displays the sequence alignment results. White and blue colors indicate the degree of comparison. The red box on the sequence indicates the position of the crRNA.

**Figure S2** Schematic of crRNA design Targeting *CpSge1* for RAA-CRISPR/Cas12a specific detection*.* The crRNA (5'-UAAUUUCUACUAAGUGUAGAUUUCCUUGGGAGCAGCAGCAG-3') was designed to precisely target *CpSge1.*
